# The Perception of “Intelligent” Design in Visual Structure

**DOI:** 10.1177/20416695221080184

**Published:** 2022-02-24

**Authors:** Filipp Schmidt

**Affiliations:** Justus Liebig University Giessen, Germany;; Center for Mind, Brain and Behavior (CMBB), University of Marburg and Justus Liebig University Giessen, Germany

**Keywords:** vision, intelligent design, shape, animacy

## Abstract

Many objects in our visual environment will appear to us either as a consequence of “intelligent” design—the purposeful action of an animal mind—or as a consequence of self-organization in response to nature's forces—for example, wind or gravity. Here, the origin of this distinction is studied by collecting human judgements about skeletal representations of objects, that reduce objects to their basic visual structure. The results suggest that humans attribute an animate origin to visual objects with basic structures exhibiting straight lines and right angles.

We perceive structure everywhere in our visual environment, in objects and scenes, no matter whether they were created by humans and other animals (Norman, 2013; Sane, Ramaswamy, & Raja, 2020) or by self-organization in response to nature's forces such as wind, water and gravity (Ball, 2016). This study is testing whether human observers can, by mere inspection of these structures, distinguish between “intelligent” design and nature's design—which is not always easy (Figure 1A, B). For this, 14 images of objects were compiled that were either created by purposeful animal minds (two each of circuit boards, streets, and beetle tunnels) or inanimate, natural forces (two each of mineral veins, neurons, roots, and rivers). Note that even though these objects can be broadly grouped into these two classes, they originate from a plethora of causes. For example, the topology of a city's streets is not only a function of transportation efficiency but also of the underlying terrain or the city's inhabitants’ culture; the morphology of rivers is not only a function of the underlying terrain but also of the vegetation on its banks or the volume and composition of sediments in its water.

**Figure 1.**
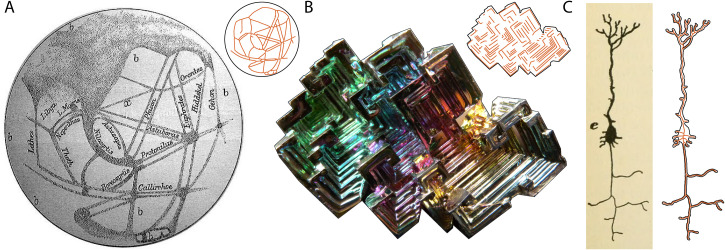
Examples of visual structures we might suspect to be of “intelligent” design, with their respective skeletons, and example stimulus creation. (A) Networks of long straight lines that were erroneously observed on Mars due to early poor-quality telescopes and illusory perception at the beginning of the 20^th^ century, sparking discussions about an alien civilization (Schiaparelli, 1888). (B) Bismuth metal exhibiting spiral, stair-stepped structures as a result of a higher growth rate at the edges compared to the interior (photograph by David Abercrombie 2010, published under Creative Commons license CC BY-SA 2.0). (C) Example stimulus creation for a mammal pyramidal nerve cell, with the corresponding manual contour drawing by the author and the superimposed skeleton in orange (original drawing by Ramón y Cajal, obtained from Howell, 1911, p. 188).

In the next step, the author created manual contour drawings inspired by these 14 images to remove irrelevant context, and then digitalized and translated these drawings into skeletons using Matlab’s (2020) *bwskel* function—which just retained the basic visual structure without any of the contour details (Figure 1C).

Observers were asked to arrange all 14 visual structures on a continuum from “animate origin” (defined as the result of purposeful planning or action of an animal mind—like a wardrobe or a bird's nest) to “inanimate origin” (defined as the result of no purposeful, inanimate forces—like a rock formation or a dune). Note that the definition and examples for animate origin explicitly included the creations of humans as well as of other animals. After completing this arrangement, observers also performed a free naming task where they indicated for each of the structures what they thought it was based on (e.g., “river” or “nerve path”). The results were twofold (summarized in Figure 2).

**Figure 2.**
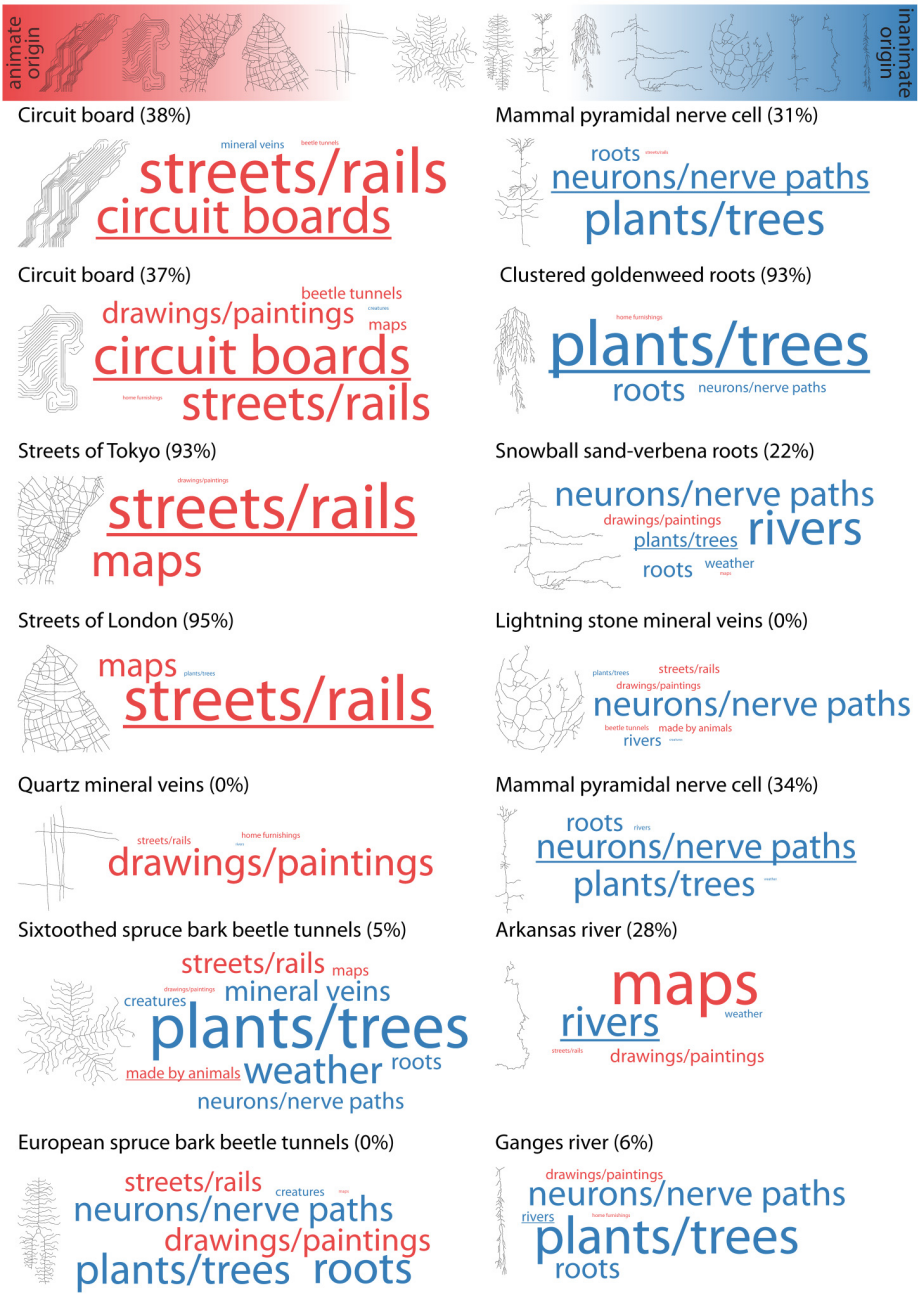
Results of ordering and free naming tasks for all visual structures (i.e., skeletons). At the top, all stimuli are ordered on the continuum between animate origin (left, red) and inanimate origin (right, blue) based on their average rank across all observers (n = 20: 18 w, 2 m, ages 19–37 years). Below, each stimulus is plotted next to a word cloud based on the frequency of the corresponding free naming responses. The percentage of correct responses for each stimulus is shown in brackets, with the correct response underlined in the word cloud. Also, all responses in the word cloud are colored according to their ground truth origin (animate, red; inanimate, blue). For example, only 34% of responses for the neuron shown in Figure 1C were correct, however, all incorrect responses were also of inanimate origin. Note that before plotting word clouds, free naming responses were organized by independent raters (n = 3: 3 w, ages 20–25 years) into categories defined by the ground truth labels (circuit boards, mineral veins etc.) or the raters themselves, and all categories with ≤ 3 responses were discarded.

First, on average human observers were pretty accurate in their arrangement with respect to animate or inanimate origin—only Quartz mineral veins were wrongly considered to be of rather animate origin compared to beetle tunnels (Figure 2, top). Second, this good accuracy was even obtained for stimuli where identification performance was low. In other words, observers were good in identifying “intelligent” design even when being bad in recognizing the thing whose structure was shown (Figure 2, bottom). For example, circuit boards were often misidentified as streets or trails (which however, are also of animate origin), and the Ganges river was often misidentified as plants or neurons (which however, are also of inanimate origin).

However, this good accuracy with respect to judgements about animate or inanimate origin was observed neither for the Quartz mineral veins—which were most often misidentified as a drawing or painting, nor for the beetle tunnels—which were most often misidentified as plants or neurons. Why do these particular mistakes happen? Previous studies measured particular attributes of shape skeletons and related them to human judgements, for example, the visual classification of objects into animals and leaves (e.g., skeletons of animal shapes, unlike those of leaves, have multiple curvy limbs; Wilder, Feldman, and Singh, 2011). The current findings might be explained following a similar rationale: specifically, observers might be more likely to judge visual structures as of animate origin if they contain more straight lines and right angles. This is also suggested by findings using texforms—synthetic stimuli that preserve curvature and basic texture information from object and animal images but render the objects and animals unrecognizable (Long, Störmer, & Alvarez, 2017): more boxy (as opposed to curvy) texforms were rather perceived as manmade objects (as opposed to animals).

In the current study, this hypothesis was confirmed by performing an additional experiment in which observers (n = 10: 4 w, 6 m, ages 23–64 years) arranged all 14 visual structures on a continuum from “straight lines and right angles” to “no straight lines and no right angles”. Indeed, the resulting ordering correlated strongly to the ordering according to animate and inanimate origin (rank correlation, *r*(12) = 0.86, p < .001). This heuristic—which is failing for Quartz mineral veins and beetle tunnels—is presumably based on the observation of the typical structure of objects and scenery in our natural environment. With rare exceptions (e.g., Figure 1B), structures that are formed by nature's forces simply do not exhibit straight lines and right angles, making these features a perceptual hallmark of “intelligent” design.
